# *EHP* Highlights of the Past Year

**DOI:** 10.1289/EHP777

**Published:** 2016-08-01

**Authors:** Sally Perreault Darney

**Affiliations:** *Environmental Health Perspectives*, National Institute of Environmental Health Sciences, National Institutes of Health, U.S. Department of Health and Human Services, Research Triangle Park, North Carolina, USA

Approaching the first anniversary of my becoming Editor-in-Chief of *EHP*, I want to provide an update to our readers and authors about recent events and changes. My first priority is to thank our associate editors and reviewers, who donate their valuable time and expertise to ensure that peer review is fair and rigorous, and who help *EHP* publish the most credible and important environmental health research. One measure of their contribution is reflected in the increase in *EHP*’s impact factor from 7.98 in 2014 to 8.44 in 2015. Over the past 12 months, *EHP* continued to receive about 120 new submissions per month. We sent about 30% forward for peer review, with two-thirds of those becoming accepted after peer review, for a final acceptance rate of about 20%.

Special thanks go out to the following associate editors who completed terms of service in 2016: Michelle Bell, Douglas W. Dockery, Bruce Fowler, Donald A. Fox, Eliseo Guallar, Lizbeth Lopez-Carillo, Chensheng (Alex) Lu, and Kyle Steenland. We have added about 20 new associate editors to balance the expertise needed for submissions in the diverse fields that make up the environmental health research community, especially in areas of growth such as epigenetics, metabolomics, climate change, and social factors. The full list of current associate editors can be found on the *EHP* website at http://ehp.niehs.nih.gov/editorial-board/.

Many, many scientists serve as peer reviewers for *EHP*. In calendar year 2015, a total of 874 reviewers completed at least one review for *EHP*. We thank them all mightily and extend special appreciation to those who went above and beyond the call of duty to review four or more papers: Sara Adar, Adrian Barnett, Xavier Basagaña, Michael Bates, David Bellinger, James Bonner, Joseph Braun, John Bucher, Jessie Buckley, Jonathan Chevrier, Marc Edwards, Suzanne Fenton, Kelly Ferguson, Francesco Forastiere, Rebecca Fry, Ulrike Gehring, Ghassan Hamra, Jaime Hart, Gerard Hoek, David Hondula, Kazuhiko Ito, Peter James, Margaret Karagas, Marianthi-Anna Kioumourtzoglou, Seth Kullman, Christopher Lau, Robert Laumbach, Johanna Lepeule, Matthew Longnecker, Thomas Luben, Thomas McKone, Hua Naranmandura, Tim Nawrot, C. Arden Pope III, Craig Rowlands, Jason Sacks, Jonathan Samet, Ellen Silbergeld, Gina Solomon, Massimo Stafoggia, David Stieb, Matthew Strickland, Adam Szpiro, Susan Teitelbaum, George Thurston, Marie Vahter, Jennifer Weuve, and Kai Zhang.

I also want to acknowledge *EHP*’s Operations Manager, Shaun Halloran. Since joining *EHP* in the spring of 2015, Shaun has brought his extensive experience in journal management and production to our team. He shepherded us through an important transition to a new manuscript processing system this past spring and helped us institute a number of features designed to improve peer-reviewer performance and efficiency. For example, we now ask reviewers to complete an online checklist to ensure that all aspects of scientific ethics, rigor, reproducibility, and transparency have been evaluated. We believe this is especially helpful for new reviewers and ensures consistency across all reviewers. We also instituted a more detailed expertise classification system to assist associate editors in finding qualified reviewers—please log in to Editorial Manager at http://www.editorialmanager.com/ehp/ and update YOUR expertise—and a new checklist for authors to make sure that all required materials accompany each submission.

In collaboration with *EHP*’s Science Editor, Jane Schroeder, we have also modified our process for preliminary review of all submissions, which includes consistent use of criteria for quality, relevance, and importance, in consultation with an associate editor when needed. Together, the goal of the *EHP* editorial team is to improve submission and peer-review processes up front and thereby obviate the need for additional revisions at the end of the process. In addition, I am very pleased to announce that we have eliminated a backlog of papers that had been awaiting a final internal review, which will substantially reduce the time to advance publication.

Given that *EHP* is fully online, we are also relaxing historically strict word limits (see *EHP*’s revised Instructions to Authors at http://www.ehponline.org/instructions-to-authors/). For each type of article, we now provide recommended word limits for the main text only, excluding references, tables, and figure legends. This change is made in the spirit of rigor and reproducibility, emphasizing the need to include in the main text key methods and results to ensure that the design, limitations, and primary conclusions are clear, while using supplemental material for background details and results of secondary analyses that in the past might have been reported as “data not shown.”

In summary, we have made peer review more effective and efficient, and have substantially reduced the time to final decision, without sacrificing careful and thorough peer review.

As I convene a new *EHP* Advisory Committee in the coming months, we will begin to address important issues that are under intense discussion in the scholarly publishing community. These include transparency and ethical issues related to open data and data access, as well as a growing interest in the online posting of preliminary manuscripts before they are submitted to a journal for peer review. Discussions about these issues at the 2016 Council of Science Editors meeting (which Shaun and I attended) stressed the need for clear guidelines and sustainable infrastructure to support such efforts.

With these and other challenges ahead, I am looking forward to another successful and productive year for *EHP* and to receiving your best papers!

